# The prototype foamy virus protease is active independently of the integrase domain

**DOI:** 10.1186/1742-4690-9-41

**Published:** 2012-05-10

**Authors:** Ralf Spannaus, Maximilian J Hartl, Birgitta M Wöhrl, Axel Rethwilm, Jochen Bodem

**Affiliations:** 1 Institut für Virologie und Immunbiologie, Universität Würzburg, Versbacher Str. 7, Würzburg, 97078, Germany; 2 Lehrstuhl für Struktur und Chemie der Biopolymere & Research Center for Biomacromolecules, Universität Bayreuth, Bayreuth, Germany

**Keywords:** Foamy virus, Regulation of protease activity, PARM, Integrase, GagPol fusion protein

## Abstract

**Background:**

Recently, contradictory results on foamy virus protease activity were published. While our own results indicated that protease activity is regulated by the viral RNA, others suggested that the integrase is involved in the regulation of the protease.

**Results:**

To solve this discrepancy we performed additional experiments showing that the protease-reverse transcriptase (PR-RT) exhibits protease activity in vitro and in vivo, which is independent of the integrase domain. In contrast, Pol incorporation, and therefore PR activity in the viral context, is dependent on the integrase domain. To further analyse the regulation of the protease, we incorporated Pol in viruses by expressing a GagPol fusion protein, which supported near wild-type like infectivity. A GagPR-RT fusion, lacking the integrase domain, also resulted in wild-type like Gag processing, indicating that the integrase is dispensable for viral Gag maturation. Furthermore, we demonstrate with a *trans*-complementation assays that the PR in the context of the PR-RT protein supports in *trans* both, viral maturation and infectivity.

**Conclusion:**

We provide evidence that the FV integrase is required for Pol encapsidation and that the FV PR activity is integrase independent. We show that an active PR can be encapsidated in *trans* as a GagPR-RT fusion protein.

## Background

Foamy viral assembly and maturation differs in many ways from the orthoretroviral counterpart. Foamy viruses (FVs) express the Pol protein from a specific transcript and not from a Gag-Pol precursor [1-6]. This leads to the expression of a separate Pol polyprotein consisting of a protease (PR), reverse transcriptase (RT) and integrase (IN) domain. Consequently, the assembly of FVs is complex since interaction of the viral RNA with Pol has been shown to be required for Pol uptake [7,8]. Several PR cleavage sites in Pol have been determined in vitro [9], but the in vivo maturation of foamy viral proteins appears to be very limited as compared to orthoretroviruses. Single cleavage sites were identified in vivo in both, Gag and Pol. Pol cleavage results in the free IN domain and a PR-RT fusion protein, whereas a p3 peptide is cleaved off from the carboxyl terminal end of Gag [10]. Despite the limited in vivo cleavage, the activity of the PR has to be tightly controlled to allow packaging of the full-length Gag and Pol precursor proteins into the virus. The PR activity is essential for viral infectivity [5]. The separate PR domain was shown to exhibit only a weak tendency to form active dimers in vitro [11,12]*.* PR dimer formation and activation are achieved, as we have shown recently, by the activation of the PR by a specific interaction of PR-RT with the viral PR activation RNA motif (PARM) [11,13]. PARM is located in the IN region of the viral genome [7,11]. Recently, Lee et al. [13] reported that PR activity requires the IN domain. Additionally, IN was suggested to be essential for dimerization of the Pol protein, which subsequently is needed for PR activity. Here, we wanted to re-analyse the Pol domains’ requirements for PR activity both in vitro and in vivo*,* using a novel approach to target Pol independently of RNA or Pol domains into FV particles.

## Results and discussion

### PR-RT exhibits PR activity in an RNA dependent way in vitro

**Figure 1:**
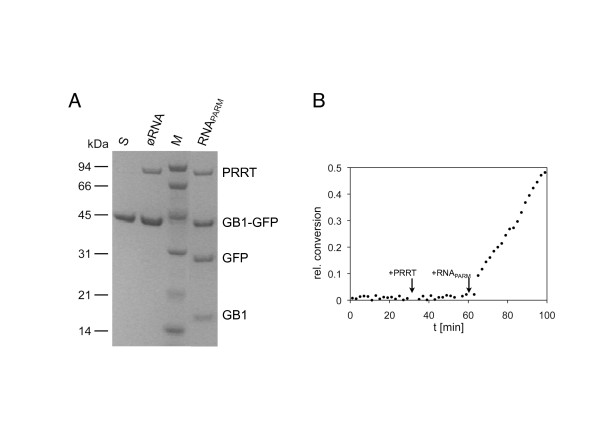
**Proteolytic activity of PFV PR-RT in vitro.** (**A**) The GB1-GFP substrate was incubated at 37°C with PFV PR-RT in the presence or absence of RNA_PARM_. Reaction products were separated on 10% BisTris gels. M: molecular weight marker, the sizes of the standard proteins are indicated on the left; S: uncleaved substrate. (**B**) For kinetic studies the conversion of the GB1-GFP substrate was observed by fluorescence anisotropy. The substrate was incubated at 37°C. PFV PR-RT and RNA_PARM_ were added subsequently after 30 and 60 min, respectively.

In two in vitro assays the ability of Prototype foamy virus (PFV) (previously known as human foamy virus) PR-RT to cleave a substrate containing the RT-IN cleavage sites between the streptococcal protein G (GB1) and the green flourescence protein (GFP) was investigated [12]. The first assay identifies the PR cleavage products after incubation of the GB1-GFP substrate with PFV PR-RT and RNA containing the PARM (RNA_PARM_) by SDS polyacrylamide gel electrophoresis (PAGE) (Figure 1A). In a second assay the conversion of the PR substrate was followed by measuring the differences in fluorescence anisotropy of the GFP moiety in the GB1-GFP fusion protein and the free GFP cleavage product after the successive addition of PFV PR-RT and RNA_PARM_ (Figure 1B). Both assays show that PR cleavage is depending on the presence of RNA_PARM_. However, PR cleavage occurs in the complete absence of IN, indicating that IN is not required for PR activity in vitro. Nevertheless, we cannot rule out that the presence of IN might lead to more stable dimers or oligomers, thus enhancing PR activity. Therefore the following in vivo experiments were conducted.

### PR-RT exhibits PR activity in vivo

**Figure 2:**
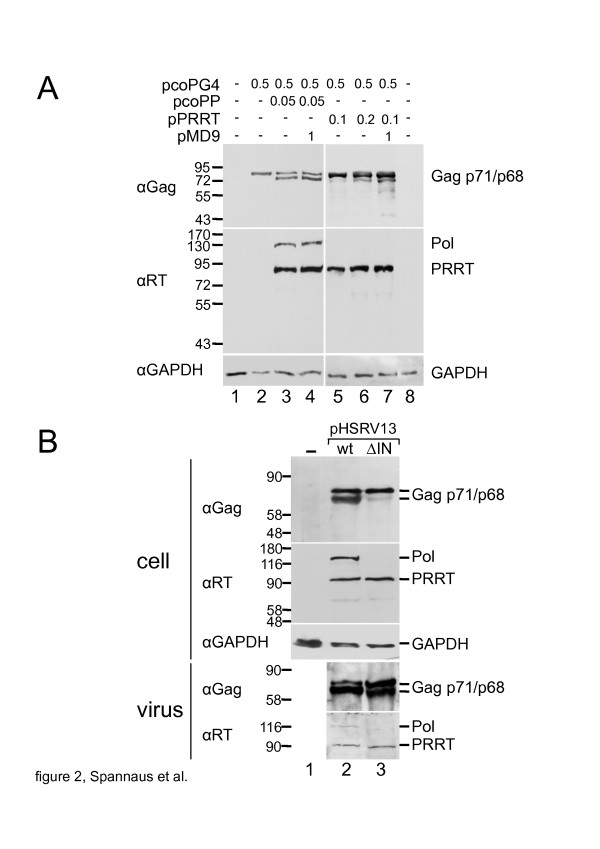
**In vivo cell culture PR-activity assays and deletion of integrase domain in the proviral context.** (**A**) HEK 293 T cells were cotransfected either with a codon-optimized PFV pcoPP or a pPR-RT expression plasmid, 0.5 μg of the pcoPG4 as PR substrate and the *gfp*-encoding pMD9 as source for viral RNA (DNA amounts are indicated in [μg]). PR activity and Pol expression was determined by Gag (upper panel) and Pol (middle panel) Western blotting analysis using monoclonal antibodies. Determination of the GAPDH concentration served as loading control (lower panel). Positions of the molecular size makers are indicated. (**B**) The IN is required for Pol encapsidation. Viruses isolated from cell culture supernatants and cellular lysates were analysed for Pol and Gag expression and maturation by Western blotting.

In order to analyse the influence of the IN domain on PR activity in vivo a stop codon was inserted downstream of the RT-IN cleavage site in a codon optimized PFV *pol* expression plasmid (pcoPP) by site directed mutagenesis leading to the pPR-RT plasmid. HEK 293 T cells were transfected with the codon optimized *gag* expression plasmid (pcoPG4), increasing pcoPP or pPR-RT amounts with or without a foamy viral vector genome encoding *gfp* (pMD9) (Figure 2A). *Pol* and *gag* expression and PR activity were analysed by Western blotting using Gag and Pol specific monoclonal antibodies [14,15]. Both, Pol as well as PR-RT, promoted Gag cleavage at the p3 cleavage site (Figure 2A). Transfection of increasing amounts of the Pol- or PR-RT-specific constructs, respectively, resulted in an increase in cleavage efficiency, which is probably due to overexpression of *pol* or PR-RT in the vector based system, as shown previously [11]. Nevertheless, cleavage of Gag or Pol was further enhanced by the expression of the vector genome, indicating the requirement of PARM for optimal PR activity [11].

Comparing both, Pol- and PR-RT-mediated Gag processing, a higher PR activity in Pol expressing cells was observed. This could be due to higher Pol expression levels (Figure 2). However, it is more likely that the somewhat lower PR activity with PR-RT was the result of a decrease of PR-RT incorporation into cell-associated or intracellular viruses, as IN is required for FV Pol encapsidation.

### The IN domain is required for pol encapsidation

To confirm this result in the context of the PFV proviral clone (pHSRV13), the complete IN domain downstream of the RT/IN cleavage site was deleted. BHK cells were transfected with the proviral plasmids pHSRV13 or pHSRV13∆IN. Supernatants were collected after two days and viruses were pelleted through a sucrose cushion. The pelleted viruses were lysed in RIPA buffer. Gag and Pol proteins were analysed by Western blotting (Figure 2B). After deletion of the IN domain some Gag cleavage was observed, (Figure 2B), leading to the conclusion that the IN might enhance the PR activity, but is not strictly required for PR activity. The lower PR-RT incorporation can be observed in the recent publication by Lee at al ([13] Figure 1D) as well, underlining our results. In addition, our IN deletion mutants showed a strong decrease in cellular PR-RT amounts. Again, this is in line with the results shown previously [13].

### In-frame expression of *gag* and *pol* gives rise to infectious virus and genome independent pol encapsidation

**Figure 3:**
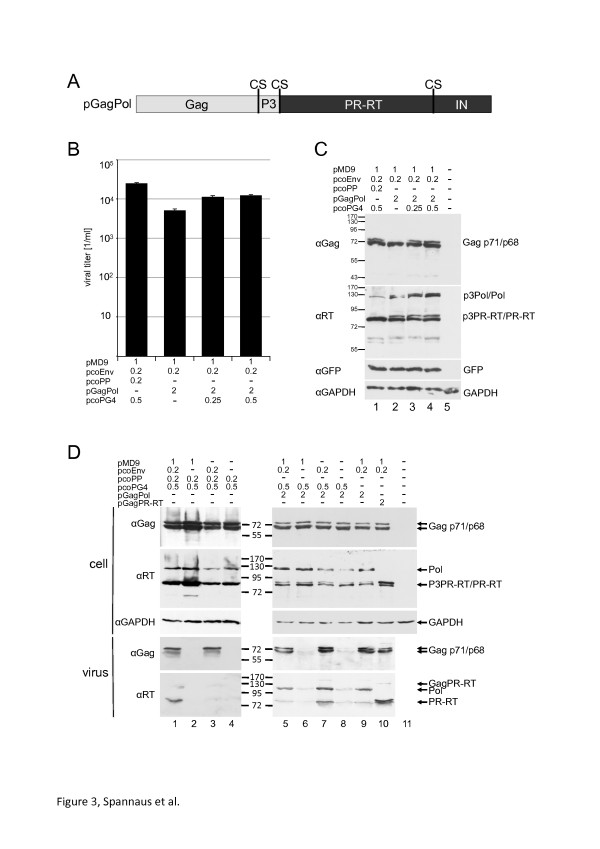
**Analysis of virus infectivity and PR activity in vivo using different GagPol-fusion proteins.** (**A**) A schematic diagram of the GagPol precursor protein is depicted on top of the panels: CS, PR cleavage site. (**B**) Comparison of the virus titer of FV codon optimized vector system (VS) [11] or the GagPol fusion plasmid cotransfected with an *env* expression construct and pMD9 as *gfp*-encoding genome. Additional amounts of a *gag* expression plasmid (pcoPG4) used for cotransfections are indicated. The transfected DNA amounts are indicated in [μg]. Transfections were performed in triplicate assays. The error bars represent the standard deviation. (**C**) Western blotting analysis of the samples from Figure 3A. The cellular and viral Gag/Pol amounts and processing using Gag and RT specific antibodies were analysed (VS: FV vector system [11]). (**D**) The virus release of GagPol fusion is Env dependent but genome independent and the processing of Gag is IN independent. Western blotting analysis of the cellular and viral Gag/Pol amounts and processing. HEK 293 T (2×10^5^) cells were transfected with pcoPE, pMD9, pcoPG4, and either pGagPol or pGagPR-RT. The transfected DNA amounts are indicated in [μg]. Analysis of the GAPDH expression served as a loading control.

To study Gag processing in viral particles in an IN independent way, we first fused the Gag and Pol reading frame, thereby coupling FV Pol uptake to Gag. A similar approach for FV Pol expression in the proviral context was published recently [16]. In doing so, we sought to show that the Pol or PR-RT uptake can take place as a GagPol fusion protein. Thus, we analysed whether FV Pol can be incorporated into particles as a Gag-Pol fusion protein and whether this results in the formation and release of infectious viral particles. In a first step the codon optimized Gag and Pol ORFs were fused and a p3-Gag cleavage site (CS) was inserted upstream of the PR-domain leading to a PFV Gag-p3CS-Pol expression plasmid called pGagPol (Figure 3A). pGagPol is devoid of any RNA sequences that have been shown to be required for PR activity. HEK 293 T cells were cotransfected with the GFP encoding pMD9 as a source for viral RNA, with the *env* expression plasmid (pcoPE), pGagPol and additionally with 0.25 or 0.5 μg of the codon optimized Gag-encoding plasmid pcoPG4 since the Gag/Pol ratio might be important for proper virus morphology (Figure 3B and C). In addition, HEK 293 T cells were cotransfected with the FV vector system (VS) consisting of pcoPE, pcoPP, pcoPG4, and pMD9 as described before [17]. The supernatants were titrated 2 days after transfection and transduction rates were determined (Figure 3B). Surprisingly, transfections of cells with pcoPE, pMD9 and the pGagPol fusion plasmid even in the absence of the additional *gag* expression plasmid led to viral titers of about 1/3 of the vector system control. Viral titers could even be raised by co-transfecting cells with increasing amounts of pcoPG4 (Figure 3B). These experiments show that the expression of a GagPol fusion protein leads to infectious FVs and a separate Pol is not required in the vector context. The Gag and Pol expression and processing of the GagPol precursor protein alone or with additional Gag was analysed by Western blotting using monoclonal antibodies against Gag and Pol as described before [18] (Figure 3C). This analysis revealed a wt-like Gag processing, whereas an additional Pol pro-duct, probably consisting of p3-PR-RT, was detected.

It was shown previously that FV Gag release is dependent on the viral Env and that Pol encapsidation requires the viral genome [19]. To further investigate whether Pol uptake is genome dependent and GagPol particle release is Env-dependent, we analysed if Env or pMD9 is required for particle release. Therefore, HEK 293 T cells were transfected as described above with either the vector system or with pGagPol in the presence or absence of pMD9, harbouring the viral genome, or pcoPE expressing Env (Figure 3D). In order to increase particle formation, the cells were cotransfected with the *gag* expression vector pcoPG4 and the pGagPol plasmid. Viruses were partially purified by ultracentrifugation through a sucrose cushion. Cellular and viral Pol and Gag processing and amounts were analysed by Western blotting (Figure 3C). This analysis revealed that more Pol was incorporated into virus particles that were derived from expressing cells as compared to cells transfected with the vector system, indicating that FVs tolerate higher Pol incorporation than seen with the wt. In contrast to the wt virus, Pol incorporation was genome-independent. In addition, Pol was not cleaved off from Gag first and subsequently incorporated in a RNA dependent way, since Pol was encapsidated even in the absence of the viral genome. (Figure 3D, compare lanes 3 (separate Pol without viral genome)) and 7 (GagPol fusion protein without viral genome). Env was required for particle release of cells transfected either with vector system or pGagPol (Figure 3D, lanes 1 & 2 and lanes 7 & 8). These experiments demonstrate that a separate Pol is not required for FV assembly and that a Gag-Pol fusion protein results in infectious viruses.

### The viral GagPR-RT fusion protein exhibits PR activity

Having established that a GagPol fusion protein leads to virus particles, a codon optimized GagPR-RT expression plasmid was created by PCR to investigate PR activity. This plasmid encodes the complete Gag, PR and RT ORFs followed by a stop codon. HEK 293 T cells were transfected with pcoPE, pMD9, pcoPG4, and either pGagPol or pGagPR-RT. Gag and Pol amounts as well as Gag processing of the harvested viruses was analysed by Western blotting (Figure 3D, lanes 9 and 10). The PR activity in the context of the codon optimized Pol constructs is not strictly dependent on the viral RNA, due to the high over-expression rate of *pol*[11]. Viruses from both, pGagPol and pGagPR-RT transfected cells, showed similar Gag processing, indicating that the presence of the IN domain is not crucial for PR activity.

### PR-RT in *trans* is sufficient for PR mediated cleavage and infectivity

**Figure 4:**
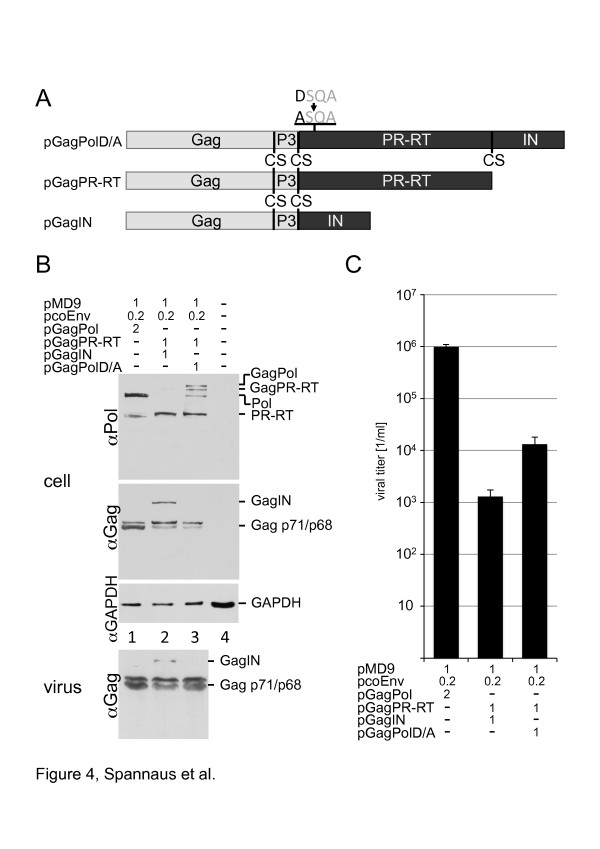
**Co-expression of a mutated GagPol fusion protein with an inactive PR domain together with a GagPR-RT fusion protein leads to both maturated Gag and Pol proteins and viral infectivity.** (**A**) Scheme of the plasmids used in this experiment. (**B**) Western blot from both transfected cells (upper three panels) and partially purified viruses (lower panel) using αGag and αPol monoclonal antibodies. HEK 293 T cells were transfected with pMD9, pcoPE and pcoPG4 and either pGagPol (lane 1) or a combination of pGagIN and pGagPR-RT (lane 2) or pGagIN and pGagPolD/A (lane 3). The transfected DNA amounts are indicated in [μg]. Determination of cellular GAPDH amounts served as loading control. (**C**) Viral titers were determined on BHK-ll indicator cells. Transfections were performed in triplicate assays. The error bars represent the standard deviation. The transfected DNA amounts are indicated in [μg].

Since expression of GagPR-RT together *env, gag* and the vector genome led to wt‒like Gag cleavage (Figure 3D), two trans-complementation assays were performed. The PR domain was expressed and encapsidated separately from the IN domain in both assays. First, we co-transfected HEK 293 T cells with the GagIN fusion together with a GagPR-RT fusion construct and with the other components of the vector system. The fused Gag domain should guarantee the encapsidation of the PR-RT and IN domains. Gag and RT expression and processing were determined in cellular lysates (Figure 4B). The Gag matu-ration was confirmed by Western blots of partially purified viruses. This experiment indicates that PR-RT is capable of cleaving the Gag precursor protein (Figure 4B). However, these transfections resulted in a significant loss of infectivity (approximately 3 logs) probably due to a failure in genome incorporation (Figure 4C).

Therefore, in a second approach the plasmid pGagPolD/A was used as IN source in the context of the Pol protein. The pGagPolD/A plasmid encodes a GagPol fusion protein with a mutation in the PR-active site (Asp24 > Ala) [5] (Figure 4A). HEK 293 T cells were co-transfected with pGagPol alone or with pGagPolD/A and pGagPR-RT, as a PR source (Figure 4A and B). Western blotting analysis showed maturation of both cellular and viral Gag proteins. Furthermore, analysis of the viral infectivity revealed that both, recombinant viruses comprising an encapsidated GagPR-RT protein were infectious (Figure 4C) although viral titers (10^4^/ml) were significantly lower than those of the wt (10^6^/ml). These experiments prove clearly that the IN domain in *cis* is not necessary for PR activity, since we show here that the FV PR in *trans* is sufficient for viral maturation.

We provide evidence that the IN domain is required  for efficient *pol* expression and for Pol encapsidation. Recapitulatory, all publications containing data on FV Gag and Pol cleavage in the presence or absence of the viral genome confirm the necessity of the viral RNA for PR activity. [11,20-22]. In addition, we show that a Gag-Pol fusion protein can give rise to infectious viruses and that the expression of a separate Pol is not required for FV infecti-vity. Furthermore, with the GagPol fusion protein we can now separate Pol encapsidation from the genome and study genome incorporation separately.

In summary, we provide evidence that the FV IN domain is not required for PR activity: First, expression of PR-RT in cells is sufficient for Gag processing. Second, the PR activity in vitro is RNA_PARM_ dependent but IN independent. Third, incorporation of PR-RT into viruses via a GagPR-RT fusion protein leads to Gag cleavage in purified viruses. Fourth, the PR activity can be encapsidated in *trans* as PR-RT molecule in the absence of the IN domain leading to mature and infectious viruses.

## Methods

### In vitro PR activity assays

To determine proteolytic activity in vitro*,* PFV PR-RT and the GB1-GFP substrate containing the RT-IN cleavage site between GB1 (immunoglobulin binding domain B1 of the streptococcal protein G) and GFP (green fluorescence protein) were purified as described previously [23-25]. In a first assay, 10 μM of the GB1-GFP substrate was incubated for 2 h at 37°C in 50 mM Na_2_HPO_4_/NaH_2_PO_4_ pH 6.4 and 100 mM NaCl with 2.5 μM PFV PR-RT in the presence or absence of 0.5 μM RNA_PARM_ in a total volume of 20 μl. Reaction products were separated by electrophoresis on 10% BisTris gels (Invitrogen, Karlsruhe, Germany) in 50 mM 2-(N-morpholino)ethanesulfonic acid buffer pH 7.3, 50 mM Tris base, 0.1% SDS and 1 mM EDTA.

In a second assay, the conversion of the GB1-GFP substrate was observed by measuring the change in fluorescence anisotropy of GFP upon cleavage of the substrate (excitation wavelength: 395 nm; emission wavelength: 517 nm) in an L-format Jobin-Yvon Horiba Fluoromax fluorimeter. Therefore, 10 μM GB1-GFP substrate was incubated at 37°C in 50 mM Na_2_HPO_4_/NaH_2_PO_4_ pH 6.4 and 100 mM NaCl. After 30 min 2.5 μM PFV PR-RT and after 60 min 0.5 μM of RNA_PARM_ were added.

### Plasmids and transfections

Codon-optimized prototypic FV *gag* (pcoPG4) [26], *pol* (pcoPP) [11], *env* (pcoPE) [27] and the *gfp* encoding vector genome (pMD9) [14] plasmids were used. *pGagPol*: In the first step the Gag/P3 cleavage site was added by PCR using the primers PolHpaIs/PolHIa, Herculase II polymerase (Stratagene) and pcoPP as template. The product was digested with BamHI and cloned into the BamHI/HpaI digested pcoPP vector. The resulting vector was denominated as pcoCSPol. Then Gag was amplified with the primers GagHIs and GagHIa. The product was digested and cloned into the *Hpa*I-site of the described pcoCSPol vector. The resulting plasmid was verified by nucleotide sequencing. *pGagPR-RT:* The Gag/P3 was added to *pol* and a stop codon was cloned at the RT/IN CS by PCR using the primers PolHpaIs and Pol∆INNota. The PCR product was cloned into the NotI/HpaI digested pcoPP vector. Then the Gag-coding sequence was added as described above. *pPR-RT:* An *AsiS*I-restriction site was introduced into pcoPP by site directed mutagenesis using the primers PolHIs and PolAsiSIa and the Pol100a and PolAsiSIs respectively. The PCR-product was digested with *BamH*I/*Xho*I and ligated into the *BamH*I/*Xho*I digested pcoPP vector. The pcoPPΔIN vector was digested with AsiSI and XhoI and the AsiSI/XhoI digested PCR product (primers: Pol100a and PolAsiSIs) was inserted. A stop codon was inserted 6 amino acid residues downstream of the PR RT-IN cleavage site (YVVN/CNTKKAI-Stop) by ligation of the INstop-oligo into the AsiSI site of pcoPP-AsiSI. *pGagPolD/A* (Asp24 > Ala): The catalytic centre of the PR was inactivated by amplifying the pcoPol vector with the primers pcoAflIIs and PolNarIa. The product was digested with AflII/NarI and ligated into the AflII/NarI digested pcoPol vector. The resulting pGagPolD/A vector was digested with HpaI and ligated with the Gag fragment amplified with the primers GagHIs and GagHpaw/ostop. *pHSRV13∆IN*: In order to delete the IN domain a *AsiS*I was introduced at position 5383–5389 of pHSRV13 by PCR mutagenesis using the primers HSRVAsiSIa and HSRVAsiSIs. In this site a stop codon was introduces by self-complementary primer pStop, resulting in a stop-codon at amino acid residue 562 of Pol.

All transfection reactions were performed using  Turbofect (Fermentas) and HEK 293 T or BHK cells as described before [11].

### Primers

PolHpaIs,       AACACCGTGACCCAGATGAACCCCCTGCAGCTGCTGCAGCC;         PolHIa,      TCTTGTCGTAGTGGAACTGGATCCGGGG;    Pol∆INNota,    AATTGCGGCCGCTTAGTTCACCACGTAGGAGCCCTGGGTGG; GagHIs,   AACGGTGGAGGGCAGTGTAGTCTGAGC; GagHIa,  AACGGCTCTGTCCCGCTGGTCGCCGCCAGAGGC;  GagHpaI∆CSa,  ACTGAATTCGTCCCGCTGGTCGCCGCCAGAGGC;    PolHIs,   ATGACCTACCTGGAAGATCCCCGG;    PolAsiSIa,     ATATCGCTCGAGTCCAGCGATCGCCTT CTTGGTGTTGCAGT TCACCACG;  PolAsiSIs,   ATACGCGATCGCTAAGCCCAACCTGGACGCCGAACTGG; Pol100a, TATTAGGAAAGGACAGTGGGAGTGG; pcoAflIIs, ATGGAAGACTTAAGGCA-GCGGC;    Pol21ANarIa,    ATGGTGGCGCCGCTGGCCCAGTGGG; pStop, TTAACTAAGTAAGGATCCTTACTTAGTTAAAT;     HSRVAsiSIa,    TATTCCGGAATATGCGATCGCTTTTTTGGTATTACAATTAACTACATAACTTCCTTGGG;    HSRVAsiSIs,    ATAGCGAT CGCAT GTAAT ACCAAAAAACCAAACCTGGATGC.

### Virus purification, infectivity assays, and western blotting analysis

To purify virus particles, cell culture supernatants were centrifuged at 474 x g to remove cells. The pre-cleared supernatant were loaded on a 20% sucrose cushion and centrifuged at 201,149 x g at 4°C for 2 h. To analyse the infectivity cell culture supernatants were titrated on BHK cells in triplicate assays. The number of GFP expressing cells was determined after two days using a fluorescence microscope. Viral titers were subsequently calculated.

Gag and Pol were detected using monoclonal  antibodies against Gag and RT as described before [11]. GAPDH amounts were visualized using an anti-GAPDH serum (Sigma-Aldrich).

## Abbreviations

GB1a, streptococcal protein G; GFP, Green fluorescence protein; IN, Integrase; PARM, Protease activation RNA motif; FV, Foamy virus; PR, Protease; HIV, Human immunodeficiency virus; PFV, Prototype foamy virus; RT, Reverse transcriptase; wt, Wild-type.

## Competing interests

The authors declare that they have no competing interests.

## Authors’ contributions

R. S. and M. J. H. conducted the experiments, A. R. contributed to the design of the study, B. M.W. and J. B. designed the study, analysed the data and wrote manuscript. All authors read and approved the final manuscript.
